# Joint association of triglyceride–glucose index and Chinese visceral adiposity index with prevalent diabetes: a cross-sectional study

**DOI:** 10.3389/fendo.2026.1799208

**Published:** 2026-06-26

**Authors:** Yuchao Yang, Xiaobing Wu, Deliang Lv, Ziyang Zhang, Fengzhu Xie, Wei Xie, Pan Ke, Hongyun Guan, Xiaoyan Wu, Zhiguang Zhao

**Affiliations:** 1 Department of Cardiovaseular and Cerebrovascular Diseases and Diabetes, Shenzhen Center for Chronic Disease Control, Shenzhen Dermatology Hospital, Shenzhen Institute of Dermatology, Shenzhen, China; 2 School of Public Health, Guangdong Medical University, Dongguan, China

**Keywords:** Chinese visceral adiposity index, diabetes, insulin resistance, joint indicator, triglyceride glucose

## Abstract

**Background:**

Diabetes mellitus is strongly associated with insulin resistance and visceral adiposity. Recent studies have demonstrated that the triglyceride–glucose index (TyG) and the Chinese visceral adiposity index (CVAI), which reflect insulin resistance and visceral adiposity, are linked to elevated odds of diabetes. However, the combined use of TyG and CVAI for identifying diabetes remains underexplored. This study aimed to investigate the relationship among TyG, CVAI, and their joint association with prevalent diabetes.

**Methods:**

This cross-sectional study included 11,495 participants from the 2023 Shenzhen Chronic Diseases and Risk Factors Surveillance Survey. A structured questionnaire survey, physical examinations, and fasting blood sample collection were conducted for all participants. Logistic regression models were used to calculate odds ratios (ORs) with 95% confidence intervals (CIs). Restricted cubic spline (RCS) was employed to assess potential nonlinear relationships (all *P* for nonlinear <0.001).

**Results:**

The study identified 831 participants with diabetes. After adjusting for confounders, TyG (OR: 4.48, 95% CI: 3.66–5.52) and CVAI (OR: 6.02, 95% CI: 3.69–10.05) were independently associated with the odds of diabetes. Nonlinear relationships were observed for both TyG and CVAI (all *P* for nonlinear <0.001). The combination of TyG and CVAI demonstrated the highest discriminative ability (AUC: 0.785, 95% CI: 0.771–0.799), outperforming TyG alone (AUC: 0.769, 95% CI: 0.754–0.785) and CVAI alone (AUC: 0.744, 95% CI: 0.729–0.759).

**Conclusion:**

Elevated TyG and CVAI levels, as well as their combined use, were significantly associated with increased odds of diabetes. These findings suggest that TyG, CVAI, and their combination may serve as simple and effective tools for diabetes risk stratification, enabling targeted prevention and management strategies for high-risk populations.

## Introduction

Diabetes mellitus has emerged as a critical global health challenge that demands urgent public attention. According to the 11th edition of the International Diabetes Federation (IDF) Diabetes Atlas (1), approximately 589 million individuals aged 20–79 worldwide were affected by diabetes in 2024, with projections estimating a rise to 853 million by 2050, particularly in East Asia, including China and Pakistan (2). The progressive worsening of diabetes is associated with a significantly elevated risk of atherosclerotic cardiovascular disease, the leading global cause of mortality and disability (3). Notably, over 90% of diabetes cases are attributed to modifiable factors such as dietary habits and sedentary lifestyles (4). Early identification of high-risk populations during the prediabetic stage, coupled with targeted interventions, holds significant potential to mitigate disease progression, alleviate economic burdens, and enhance public health outcomes (5).

The pathogenesis of diabetes is closely linked to insulin resistance (IR) and visceral adiposity (6). While imaging modalities like computed tomography (CT) and magnetic resonance imaging (MRI) provide precise assessments of fat distribution and the hyperinsulinemic–euglycemic clamp remains the gold standard for quantifying IR, their clinical utility is limited by invasiveness, cost, and accessibility (7). Consequently, recent research has focused on identifying cost-effective and non-invasive surrogate markers. Emerging indices, such as the triglyceride–glucose (TyG) index (8) and Chinese visceral adiposity index (CVAI) (9), have demonstrated promise in identifying individuals with diabetes, offering practical alternatives for large-scale screening and early intervention strategies.

The TyG index, derived from fasting plasma glucose (FPG) and triglyceride (TG) levels, has been validated as an effective non-invasive marker for assessing metabolic health and insulin resistance (10). Concurrently, the CVAI, a population-specific metric developed based on visceral adiposity parameters (e.g., waist circumference (WC), body mass index (BMI), TG, and high-density lipoprotein cholesterol (HDL)), offers enhanced accuracy in evaluating visceral fat accumulation among Chinese adults (11). While both indices independently correlate with the odds of diabetes, their combined utility for assessing the odds of diabetes remains underexplored (12). This study aimed to elucidate the individual and synergistic associations of TyG and CVAI with prevalent diabetes, providing insights into their potential as cost-effective tools for early risk stratification.

## Methods

### Study design and participants

**Figure 1:**
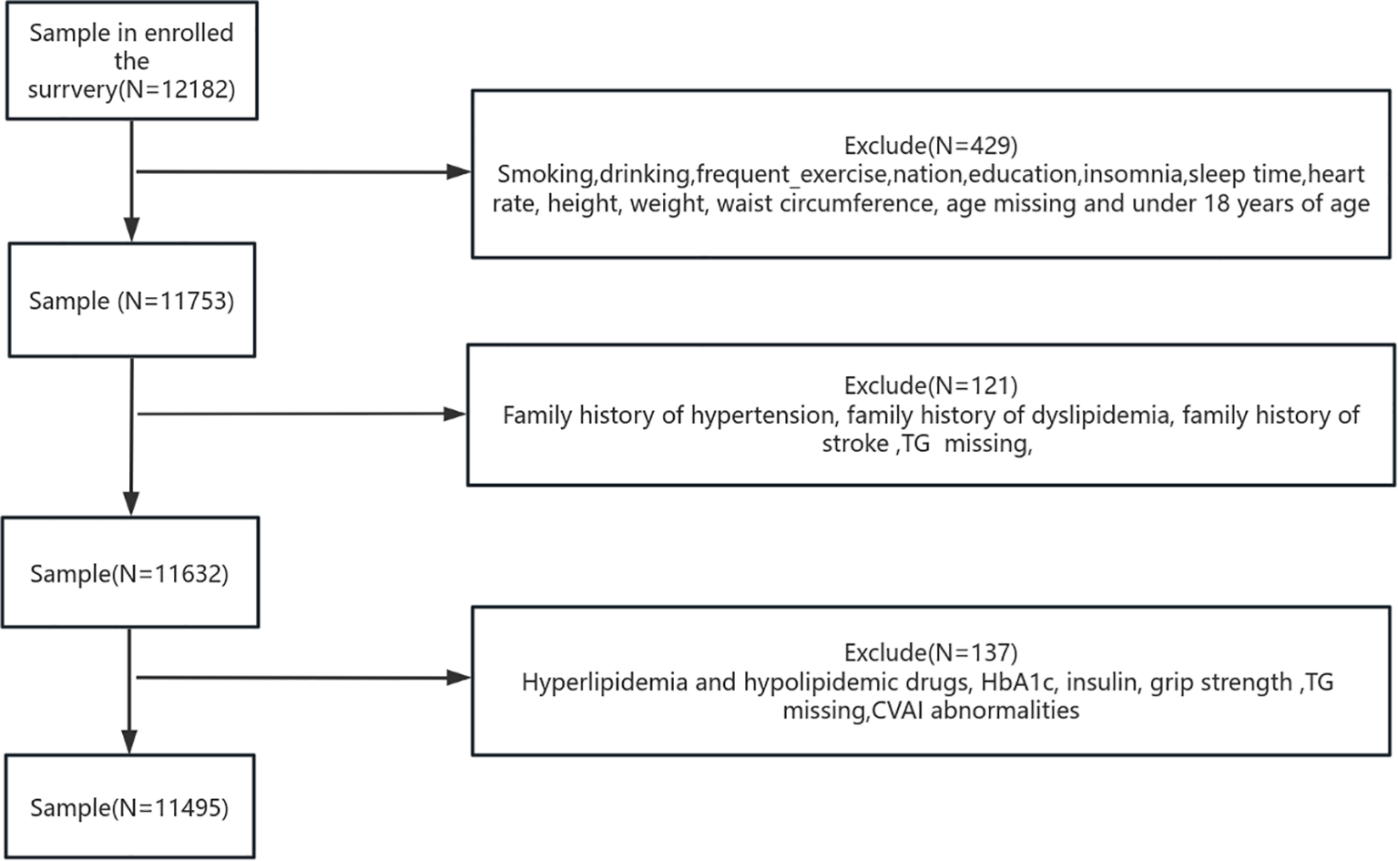
Flow chart of the selection of samples.

This cross-sectional study enrolled 11,495 participants aged ≥18 years from the 2023 Shenzhen Chronic Diseases and Risk Factors Surveillance Survey. The detailed inclusion and exclusion process is shown in **Figure 1**. A multistage stratified random sampling method was employed to ensure representative recruitment across Shenzhen. Data collection included age, gender, education level, nationality, marital status, annual household income, self-reported sleep duration, insomnia frequency, smoking status, alcohol consumption, physical activity level, weight change over the past year, and family history of hypertension, diabetes, coronary heart disease, or diabetes. hypertension, dyslipidemia, and use of antihypertensive or lipid-lowering medications, BMI, grip strength, WC, weight, height, systolic blood pressure (SBP), diastolic blood pressure (DBP), resting heart rate, TG, total cholesterol (TC), HDL, low-density lipoprotein cholesterol (LDL), FPG, glycated hemoglobin (HbA1c), and insulin levels.

### Definition of indicators

Hypertension was defined as an average systolic blood pressure ≥140 mmHg or diastolic blood pressure ≥90 mmHg across three consecutive measurements (13). Dyslipidemia was confirmed by either clinical diagnosis with current use of lipid-regulating medications (primarily statins or fibrates) or meeting any of the following serum lipid criteria: TC ≥6.22 mmol/L, LDL ≥4.14 mmol/L, TG ≥2.26 mmol/L, or HDL ≥1.55 mmol/L or <1.04 mmol/L (14). Diabetes mellitus was diagnosed based on FPG ≥7.0 mmol/L (126 mg/dL) or HbA1c ≥6.5% (15). The questionnaire used in this study did not differentiate between specific subtypes of diabetes mellitus. For the purposes of this study, diabetes mellitus was defined in participants as either a self-reported prior physician diagnosis of diabetes mellitus or current treatment with anti-diabetic agents. Ethical approval was obtained from the Institutional Review Board of Shenzhen Center For Chronic Disease Control, and written informed consent was provided by all participants prior to enrollment. TyG and CVAI were calculated using the following formulas (16, 17):


TyG=Ln[TG(mg/dl)×FPG(mg/dl)×0.5]

$$CVAI\;(male)=-267.93+0.68\times age(years)+0.03\times BMI(kg/m^{2})+4.00\times WC(cm)+22.00\times Lg\;TG(mmol/L)-16.32\times HDL(mmol/L)$$

$$CVAI\;(\text{fe}male)=-187.32+1.71\times age(years)+4.23\times BMI(kg/m^{2})+1.12\times WC(cm)+39.76\times \mathrm{lg}TG(mmol/L)-11.66\times HDL(mmol/L)$$

### Statistical analysis

Continuous variables with normal distributions were expressed as means ± standard deviations (SD) and compared between groups using independent-samples *t*-tests. Non-normally distributed variables were reported as median (interquartile range, IQR) and analyzed using the Mann–Whitney *U*-test. Categorical variables were summarized as frequencies (%) and assessed using Pearson’s chi-square (*χ*²*)* tests, with Fisher’s exact test applied for cells with expected counts <5.

The participants were categorized into subgroups based on the TyG index median value, the CVAI index quartiles group, and the combined CVAI and TyG group. Specifically, the participants were divided into eight groups (two TyG median × four CVAI quartiles). To ensure sufficient statistical power and clinical interpretability and based on the observed risk gradients, we consolidated these eight groups into four clinically relevant categories for the final analysis, as presented in **Table 1**. Multivariable logistic regression models evaluated associations between these indices and the odds of diabetes, adjusting for demographic (age, gender, and education), lifestyle (smoking, alcohol use, and physical activity), and clinical covariates (BMI, hypertension, and dyslipidemia). Model 1 adjusted for age and gender, model 2 adjusted for age, gender, education levels, marital status, smoking status, drinking status, and BMI, and model 3 adjusted for factors in model 2 and history of hypertension, dyslipidemia, frequent exercise, hypertensive drug, lipid drug, SBP, and DBP. Results were reported as adjusted odds ratios (ORs) with 95% confidence intervals (CIs).

**Table 1:** Odds of prevalent diabetes stratified by the joint of CAVI and TyG.

Indices	Case	Crude		Model 1		Model 2		Model 3	
		OR (95% CI)	*P*-value	OR (95% CI)	*P*-value	OR (95% CI)	*P*-value	OR (95% CI)	*P*-value
TyG < 8.61									
CVAI < 45.03	14 (0.56%)	Ref		Ref		Ref		Ref	
45.03 ≤ CVAI < 79.89	34 (1.94%)	3.65 (2.00–7.03)	<0.001	1.84 (0.98–3.61)	0.065	2.13 (1.07–4.46)	0.038	2.14 (1.06–4.53)	0.040
79.89 ≤ CVAI < 111.27	45 (4.80%)	9.19 (5.16–17.41)	<0.001	2.98 (1.58–5.96)	0.001	3.97 (1.75–9.45)	0.001	3.71 (1.60–9.05)	0.003
111.27 ≤ CVAI	30 (6.05%)	11.45 (6.14–22.41)	<0.001	2.64 (1.28–5.67)	0.010	4.25 (1.43–13.06)	0.010	3.78 (1.22–12.04)	0.023
TyG ≥ 8.61									
CVAI < 45.03	15 (4.36%)	8.19 (3.90–17.32)	<0.001	7.98 (3.77–17.00)	<0.001	8.02 (3.79–17.09)	<0.001	7.73 (3.65–16.46)	<0.001
45.03 ≤ CVAI < 79.89	64 (5.69%)	10.85 (6.25–20.23)	<0.001	6.72 (3.84–12.58)	<0.001	6.27 (3.56–11.82)	<0.001	6.24 (3.53–11.79)	<0.001
79.89 ≤ CVAI < 111.27	213 (11.00%)	22.22 (13.40–40.12)	<0.001	9.26 (5.51–16.86)	<0.001	8.13 (4.69–15.18)	<0.001	7.81 (4.49–14.64)	<0.001
111.27 ≤ CVAI	416 (17.50%)	38.12 (23.22–68.36)	<0.001	11.91 (7.10–21.69)	<0.001	9.57 (5.34–18.66)	<0.001	9.06 (4.92–17.75)	<0.001

Model 1 adjusted for age and gender, model 2 adjusted for age, gender, education levels, marital status, smoking status, drinking status, and BMI, and model 3 adjusted for the factors in model 2 and history of hypertension, dyslipidemia, frequent exercise, hypertensive drug, lipid drug, SBP, and DBP.

Nonlinear relationships were explored using restricted cubic splines (RCS) with three knots (*P* for nonlinearity <0.05). Receiver operating characteristic (ROC) curves assessed the discriminative capacity of CVAI, TyG, and their combination for identifying prevalent diabetes, with the area under the curve (AUC) compared using the test of DeLong. Subgroup analyses stratified by gender, physical activity status, and alcohol consumption were performed to evaluate effect modification via likelihood ratio tests. To assess the robustness of the primary findings, we conducted a series of sensitivity analyses. All analyses were conducted using R Statistical Software (V4.3.3; R Foundation). *P*-values <0.05 were considered statistically significant.

## Results

### Baseline characteristics by CVAI and TyG index levels

Participants with elevated CVAI and TyG indices exhibited higher proportions of male sex, lower educational levels, married status, smoking, and dyslipidemia. Concurrently, increasing CVAI and TyG levels were positively correlated with age, height, weight, waist circumference, BMI, HbA1c, FPG, LDL, and TG levels while inversely associated with HDL (**Table 2**).

**Table 2:** Baseline characteristics according to CVAI and TyG.

Characteristics	Total	CVAI index				*P*-value	TyG index		*P*-value
		CVAI < 45.03	45.03 ≤ CVAI < 79.89	79.89 ≤ CVAI < 111.27	111.27 ≤ CVAI		TyG < 8.61	TyG ≥ 8.61	
*N*	11,495	2,874	2,874	2,874	2,873		5746	5749	
Gender (%)						<0.001			<0.001
Male	5,373 (46.7)	737 (25.6)	1,089 (37.9)	1,557 (54.2)	1,990 (69.3)		2,038 (35.5)	3,335 (58.0)	
Female	6,122 (53.3)	2,137 (74.4)	1,785 (62.1)	1,317 (45.8)	883 (30.7)		3,708 (64.5)	2,414 (42.0)	
Age. years (mean (SD))	45.48 (12.74)	35.55 (8.82)	44.75 (10.48)	49.42 (11.52)	52.18 (13.04)	<0.001	43.25 (12.76)	47.70 (12.33)	<0.001
Education level (%)						<0.001			<0.001
Secondary school and below	7,135 (62.1)	1,273 (44.3)	1,746 (60.8)	2,021 (70.3)	2,095 (72.9)		3,268 (56.9)	3,867 (67.3)	
College and above	4,360 (37.9)	1,601 (55.7)	1,128 (39.2)	853 (29.7)	778 (27.1)		2,478 (43.1)	1,882 (32.7)	
Marital status (%)						<0.001			<0.001
Married	9,207 (80.1)	1,898 (66.0)	2,393 (83.3)	2,436 (84.8)	2,480 (86.3)		4,443 (77.3)	4,764 (82.9)	
Others	2,288 (19.9)	967 (34.0)	481 (16.7)	438 (15.2)	393 (13.7)		1,303 (22.7)	985 (17.1)	
Smoke (%)						<0.001			<0.001
Yes	2,454 (21.3)	334 (11.6)	478 (16.6)	674 (23.5)	968 (33.7)		801 (13.9)	1,653 (28.8)	
No	9,041 (78.7)	2,540 (88.4)	2,396 (83.4)	2,200 (76.5)	1,905 (66.3)		4,945 (86.1)	4,096 (71.2)	
Drink (%)						<0.001			<0.001
Yes	5,841 (50.8)	1,385 (48.2)	1,364 (47.5)	1,468 (51.1)	1,624 (56.5)		2,707 (47.1)	3,134 (54.5)	
No	5,654 (49.2)	1,489 (51.8)	1,510 (52.5)	1,406 (48.9)	1,249 (43.5)		3,039 (52.9)	2,615 (45.5)	
Hypertension (%)						<0.001			<0.001
Yes	3,650 (31.8)	674 (23.5)	597 (20.8)	949 (33.0)	1,430 (49.8)		1,544 (26.9)	2,106 (36.6)	
No	7,845 (68.2)	2,200 (76.5)	2,277 (79.2)	1,925 (67.0)	1,443 (50.2)		4,202 (73.1)	3,643 (63.4)	
Dyslipidemia (%)						<0.001			<0.001
Yes	4,703 (40.9)	359 (12.5)	927 (32.3)	1,510 (52.5)	1,907 (66.4)		1,092 (19.0)	3,611 (62.8)	
No	6,792 (59.1)	2,515 (87.5)	1,947 (67.7)	1,364 (47.5)	966 (33.6)		4,654 (81.0)	2,138 (37.2)	
Height, cm (mean (SD))	161.83 (8.35)	160.13 (7.34)	160.58 (7.83)	162.13 (8.53)	164.49 (8.90)	<0.001	160.71 (7.99)	162.95 (8.55)	<0.001
Weight, kg (mean (SD))	63.77 (12.10)	53.40 (6.42)	60.02 (7.58)	65.76 (8.45)	75.90 (11.94)	<0.001	59.46 (10.47)	68.08 (12.08)	<0.001
WC, cm (mean (SD))	82.30 (10.43)	70.52 (4.90)	78.63 (4.72)	85.12 (4.66)	94.95 (6.86)	<0.001	77.77 (9.48)	86.83 (9.31)	<0.001
BMI, kg/m^2^ (mean (SD))	24.25 (3.54)	20.81 (1.95)	23.24 (2.10)	24.97 (2.14)	27.97 (3.20)	<0.001	22.96 (3.24)	25.53 (3.36)	<0.001
HbA1c, mmol/L (mean (SD))	5.66 (0.87)	5.31 (0.51)	5.53 (0.73)	5.77 (0.89)	6.05 (1.06)	<0.001	5.43 (0.48)	5.90 (1.08)	<0.001
FPG, mmol/L (mean (SD))	5.48 (1.32)	5.02 (0.87)	5.30 (0.98)	5.61 (1.35)	6.01 (1.68)	<0.001	5.10 (0.51)	5.87 (1.70)	<0.001
TC, mmol/L (mean (SD))	5.20 (1.06)	4.81 (0.90)	5.23 (1.01)	5.40 (1.08)	5.36 (1.11)	<0.001	4.92 (0.93)	5.49 (1.09)	<0.001
HDL, mmol/L (mean (SD))	1.41 (0.31)	1.55 (0.30)	1.47 (0.31)	1.37 (0.27)	1.25 (0.25)	<0.001	1.51 (0.31)	1.31 (0.28)	<0.001
LDL, mmol/L (mean (SD))	3.33 (0.78)	2.98 (0.67)	3.35 (0.73)	3.51 (0.79)	3.47 (0.79)	<0.001	3.11 (0.70)	3.55 (0.79)	<0.001
TG, mmol/L (mean (SD))	1.70 (1.52)	0.95 (0.42)	1.38 (0.95)	1.91 (1.47)	2.57 (2.14)	<0.001	0.92 (0.24)	2.48 (1.84)	<0.001

BMI, body mass index; HbA1c, glycated hemoglobin; FPG, fasting blood glucose; TC, total cholesterol; HDL, high-density lipoprotein cholesterol; LDL, low-density lipoprotein cholesterol; TG, triglyceride; TyG, triglyceride–glucose; CVAI, Chinese visceral adiposity index.

### Diabetes incidence and independent associations

**Figure 2:**
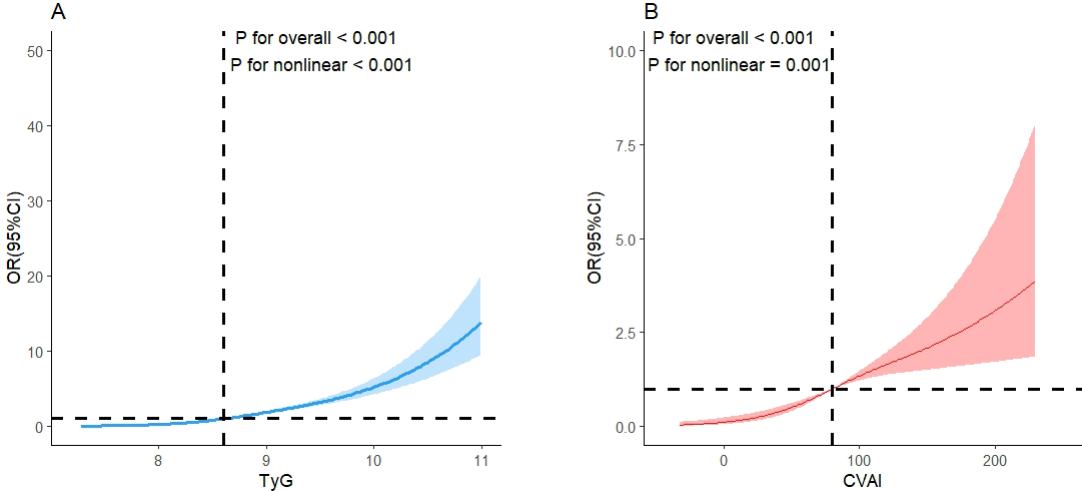
Nonlinear associations of CVAI and TyG with prevalent diabetes. **(A, B)** Graphs showing ORs for diabetes risk based on logistic regression model 3 (adjusted for age, gender, education level, marital status, nation, BMI, SBP, DBP, heart rate (HR), weight change, current smoking, current alcohol use, frequent exercise, grip strength, and medical history: dyslipidemia, hypertension, antihypertensive medication use, lipid-lowering medication use, family history of diabetes, insulin (INS), and HbA1c). Solid lines indicate ORs. Shadow shapes indicate 95% CIs.

Among 11,495 participants, 831 (7.23%) were diagnosed with diabetes. As shown in **Table 3**, the diabetes prevalence escalated with higher TyG and CVAI levels: 12.28% in the high TyG group (≥8.61) and 15.52% in the highest CVAI quartile (Q4: ≥111.27). In fully adjusted models (model 3), both indices independently associated with the odds of diabetes: TyG: OR = 4.48 (95% CI: 3.66–5.52, *P* < 0.001), CVAI (Q4:Q1): OR = 6.02 (95% CI: 3.69–10.05, *P* < 0.001), corresponding to 348% and 502% higher odds, respectively. Restricted cubic spline analysis confirmed linear associations between CVAI and TyG and odds of prevalent diabetes (CVAI: *P* for non-linear <0.001; TyG: *P* for non-linear <0.001; **Figure 2**).

**Table 3:** Odds of prevalent diabetes stratified by CVAI and TyG in baseline.

Indices	Case	Crude		Model 1		Model 2		Model 3	
		OR (95% CI)	*P*-value	OR (95% CI)	*P*-value	OR (95% CI)	*P*-value	OR (95% CI)	*P*-value
Total	831 (7.22%)								
TyG									
TyG < 8.61	125 (2.17%)	Ref		Ref		Ref		Ref	
TyG ≥ 8.61	706 (12.28%)	6.30 (5.21–7.68)	<0.001	5.09 (4.18–6.23)	<0.001	4.63 (3.79–5.70)	<0.001	4.48 (3.66–5.52)	<0.001
CVAI									
CVAI<45.03	29 (1.01%)	Ref		Ref		Ref		Ref	
45.03 ≤ CVAI < 79.89	98 (3.41%)	3.46 (2.31–5.35)	<0.001	1.98 (1.31–3.07)	0.002	2.00 (1.31–3.14)	0.002	2.01 (1.31–3.17)	0.002
79.89 ≤ CVAI < 111.27	258 (8.98%)	9.68 (6.69–14.56)	<0.001	4.09 (2.78–6.23)	<0.001	4.22 (2.76–6.66)	<0.001	4.04 (2.63–6.40)	<0.001
111.27 ≤ CVAI	446 (15.52%)	18.02 (12.57–26.92)	<0.001	6.12 (4.16–9.33)	<0.001	6.47 (4.00–10.74)	<0.001	6.02 (3.69–10.05)	<0.001

Model 1 adjusted for age and gender, model 2 adjusted for age, gender, education levels, marital status, smoking status, drinking status, and BMI, and model 3 adjusted for factors in model 2 and history of hypertension, dyslipidemia, frequent exercise, hypertensive drug, lipid drug, SBP, and DBP.

### Joint effects of CVAI and TyG

When stratified by combined CVAI and TyG categories (reference: TyG <8.61 and CVAI <45.03), the group with the highest odds (TyG ≥8.61 and CVAI ≥111.27) had a diabetes prevalence of 17.50% and an adjusted OR of 9.06 (95% CI: 4.92–17.75, *P* < 0.001), representing an 806% increased odds compared to the reference (**Table 1**).

### Subgroup and sensitivity analyses

Subgroup analyses revealed consistent associations across demographic and lifestyle strata (**Table 4**). Significant interactions were observed for marital status (*P* for interaction: 0.021) and dyslipidemia (*P* for interaction: 0.009). Notably, the combined CVAI and TyG effect remained robust irrespective of smoking, drinking, hypertension, physical activity, or insomnia status (all *P* for interaction <0.05). To evaluate the robustness of the results, we performed several sensitivity analyses, namely: (1) Participants who were taking antidiabetic, antihypertensive, or lipid-lowering medications were excluded, and the models were re-estimated (**Supplementary Table 1**); (2) TyG and CVAI were re-entered into the models as continuous variables instead of categorical ones (**Supplementary Table 2**); and (3) After excluding extreme values of BMI, TG, and FPG, all analyses were repeated (**Supplementary Table 3**). The results of these sensitivity analyses were consistent with the primary findings, supporting their robustness.

**Table 4:** Subgroup analysis of the joint association of CVAI and TyG with the odds of diabetes.

Subgroup	Case/total	TyG < 8.61 CVAI < 45.03	TyG ≥ 8.61 45.03 ≤ CVAI < 79.89	TyG ≥ 8.61 111.27 ≤ CVAI	*P* for interaction
Gender					0.077
Male	311/2,731	Ref	2.45 (0.77–9.59)	4.47 (1.19–20.01)	
Female	183/3,300	Ref	4.31 (1.40–14.10)	6.46 (1.22–36.16)	
Education level					0.438
Elementary school and below	376/3,470	Ref	4.60 (1.86–12.22)	9.20 (2.93–30.62)	
College and above	118/2,561	Ref	1.72 (0.44–7.45)	3.13 (0.45–23.57)	
Marital status					0.021
Married	429/4,649	Ref	1.81 (0.20–20.04)	4.50 (0.33–73.64)	
Others	65/1,382	Ref	4.39 (1.95–10.42)	8.31 (2.85–25.18)	
Smoking					0.249
Yes	151/1,325	Ref	0.85 (0.18–4.88)	2.73 (0.46–19.65)	
No	343/4,706	Ref	3.99 (1.64–10.27)	5.12 (1.54–17.72)	
Drinking					0.260
Yes	229/3,119	Ref	3.60 (1.06–14.72)	8.29 (1.90–42.22)	
No	265/2,912	Ref	4.10 (1.51–11.83)	5.95 (1.52–24.31)	
Hypertensive					0.217
Yes	286/2,018	Ref	4.24 (1.26–16.14)	6.44 (1.50–30.82)	
No	208/4,013	Ref	2.89 (1.06–8.43)	6.03 (1.43–26.61)	
Dyslipidemia					0.009
Yes	347/2,523	Ref	0.65 (0.23–2.09)	1.32 (0.39–4.94)	
No	147/3,508	Ref	0.58 (0.17–2.11)	1.55 (0.22–11.65)	
Exercise					0.214
Yes	420/5,033	Ref	4.55 (1.96–11.30)	9.09 (3.09–28.18)	
No	74/998	Ref	1.65 (0.25–12.00)	1.50 (0.12–20.69)	
Insomnia					0.831
Yes	357/4,109	Ref	3.42 (1.35–9.30)	6.38 (1.94–22.13)	
No	137/1,922	Ref	4.68 (1.28–19.28)	7.61 (1.29–49.14)	

Adjusted for age, gender, education level, marital status, smoke, drink, BMI, history of hypertension, dyslipidemia, exercise, use of antihypertensive medications, lipid-lowering medications, systolic blood pressure (SBP), diastolic blood pressure (DBP), waist, TG, weight, height, annual total income, nationality, sleep time, heart rate, TC, insomnia, weight change, family history of hypertension, family history of diabetes, family history of coronary heart disease, family history of diabetes, insulin levels, grip strength, HbA1, FPG, HDL, and LDL.

### Discriminative ability of TyG and CVAI for prevalent diabetes

**Figure 3:**
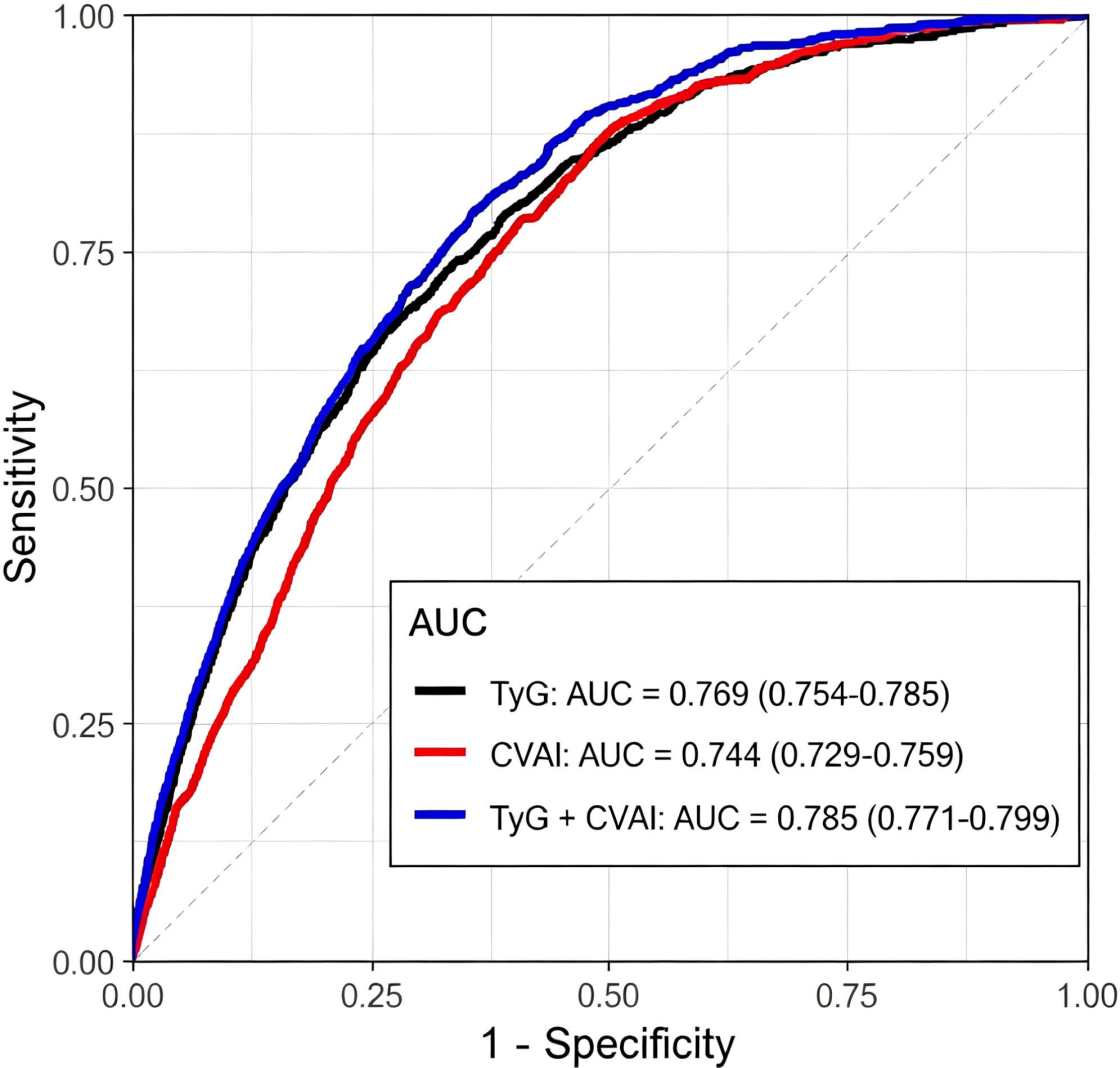
Receiver operating characteristic curves for CVAI, TyG, and CVAI + TyG in identifying prevalent diabetes based on model 3.

Based on model 3, the ROC curves indicated that the combination of TyG and CVAI had the highest discriminative ability for prevalent diabetes (AUC: 0.785, 95% CI 0.771–0.799), followed by TyG alone (AUC: 0.769, 95% CI: 0.754–0.785) and CVAI alone (AUC: 0.744, 95% CI: 0.729–0.759; **Figure 3**).

## Discussion

This study demonstrated robust associations between diabetes risk and both individual TyG index and CVAI, consistent with prior evidence (18). However, the combined discriminative capacity of TyG and CVAI for identifying diabetes remains underexplored, particularly in younger populations (19). While existing research has predominantly focused on older adults, our investigation recruited participants through multistage stratified random sampling across Shenzhen, providing unique insights into a younger, geographically representative urban population (20). This methodological approach strengthens the generalizability of our findings to working-age demographics and helps address a notable gap in current literature.

Consistent with previous studies, we observed that an elevated TyG was associated with increased odds of diabetes. Li et al. (21) in 2020 calculated the cohort study of 201,298 non-diabetic Chinese adults (aged ≥20 years) from a national health screening program and revealed that elevated TyG index independently predicted incident diabetes over a median follow-up of 3.12 years. After multivariable adjustment, each unit increase in TyG was associated with a 2.34-fold higher diabetes risk (HR: 3.34, 95% CI: 3.11–3.60). Subgroup analyses indicated stronger associations among younger individuals (*P*-interaction <0.05). Rong et al. (22) found that among 862 elderly Chinese male patients followed for 20 years, baseline TyG was associated with diabetes incidence (HR: 1.53, 95% CI: 1.29–1.80), although OGTT−derived glucose measures outperformed TyG. Extending these findings to a Japanese population, in 2023, Kuang et al. showed that a longitudinal study of 15,464 Japanese adults (mean follow-up: 6.13 years) identified a diabetes incidence rate of 39.88 per 10,000 person-years. Multivariable Cox models confirmed TyG as a significant predictor (standardized HR: 1.28, 95% CI: 1.12–1.45), underscoring its utility across diverse Asian populations. Moreover, analyses of CHARLS data by Li et al. (23) confirmed a linear relationship between TyG and diabetes risk (HR: 1.75, 95% CI: 1.56–1.97) in individuals aged ≥45 years, independent of classical confounders, and Zeng et al. (24) reported a positive association between TyG and gestational diabetes in pregnant women using NHANES data (OR: 3.43, 95% CI: 1.20–9.85). In our study, subgroup analyses indicated stronger associations among married individuals and those with dyslipidemia (*P-*interaction <0.05). Importantly, because our study is cross-sectional, these subgroup differences represent patterns of association rather than causal relationships and may be subject to selection or confounding biases—for instance, when using the lowest TyG and CVAI category (TyG < 8.61 and CVAI < 45.03) as the reference, the combination of high TyG (≥8.61) and intermediate CVAI (45.03 ≤ CVAI < 79.89) was not significantly associated with the outcome, and in the subgroup with no diagnosed dyslipidemia, the highest joint category (TyG ≥ 8.61 and CVAI ≥ 111.27) overlapped with the dyslipidemia subgroup results. These observations likely reflect the limitations of a single cross−sectional assessment and should be interpreted cautiously.

Regarding CVAI, Liu et al. (25) reported a prediabetes/diabetes prevalence of 80.53% in a cross−sectional analysis of 10,090 participants and found stronger associations of cardiometabolic indices (including CVAI) with dysglycemia in female than male patients. In longitudinal analyses, Bi et al. (26) demonstrated that CVAI independently predicted carotid plaque development (HR: 1.53, 95% CI: 1.48–1.59) in 23,522 adults. Fu et al. (9) used trajectory modeling in the China-PAR project (*n* = 52,394) and showed that higher CVAI trajectories conferred stepwise elevations in type 2 diabetes risk (e.g., high-increasing vs. low-increasing trajectory, OR: 5.50, 95% CI: 4.67–6.47), with effect modification by age, smoking, and physical activity. Similarly, Jin et al. (27) observed that among hypertensive adults aged ≥45 years, the highest CVAI quartile was associated with a 2.38-fold higher diabetes risk (HR: 3.38, 95% CI: 1.76–6.50). Our results align with these findings and further suggest that the discriminative value of CVAI may be particularly evident in subgroups with elevated TyG, possibly because CVAI helps circumvent the “obesity paradox” sometimes seen in insulin-resistant populations.

To the best of our knowledge, few studies have combined CVAI and TyG to identify prevalent diabetes. This enhanced performance may be attributed to three key mechanisms: firstly, joint assessment captures both insulin resistance (TyG) and visceral adiposity (CVAI), offering a more holistic metabolic evaluation; secondly, the combined model identifies subtle interactions between biomarkers that may precede clinical diabetes; and thirdly, integration accommodates individual variations in biomarker patterns that single indices may overlook.

In fully adjusted logistic regression (model 3), the joint high-risk group (TyG ≥ 8.6 and CVAI ≥ 111.27) yielded the highest odds ratio and was the only subgroup to show a statistically significant association with diabetes (OR: 3.74, 95% CI: 2.91–4.81). Non-significance in other groups may reflect limited power rather than absence of association as confidence intervals approached unity. Notably, CVAI demonstrated a stronger association in high-TyG subgroups, potentially due to its ability to circumvent the “obesity paradox” common in insulin-resistant populations. The dual-high group (elevated TyG and CVAI) exhibited the greatest odds of diabetes, consistent with the synergistic effects of insulin resistance and visceral adiposity. Critically, the combined index remained significantly associated even in ostensibly low-risk subgroups (e.g., non-drinkers, no baseline dyslipidemia diseases, no insomnia), and some studies show that combining novel biomarkers such as TyG and CVAI with machine learning algorithms may further improve risk stratification.

However, our study has several limitations that warrant consideration. Firstly, the cross-sectional design precludes causal inference between biomarker patterns and diabetes onset. Secondly, the exclusion of participants with missing or invalid biomarker data may introduce selection bias. Thirdly, dynamic changes in CVAI and TyG during follow-up were not captured. Fourthly, comparative performance against established anthropometric indices (e.g., BMI, WC) was not assessed. Fifthly, generalizability may be limited to Chinese populations; external validation is needed. Diabetes diagnosis relied on self-report without confirmatory tests (e.g., HbA1c, OGTT). Sixth, we did not adjust for multiple comparisons in our DeLong tests for AUCs. Given three pairwise comparisons, the Bonferroni-corrected significance threshold would be *P <*0.0167. Applying this correction, the observed difference between the combined model and TyG alone would not meet the stringent significance criterion. Therefore, the reported improvement in AUC should be interpreted as a trend rather than a definitive statistical superiority, and future larger studies are needed to confirm this incremental discriminative value. Finally, our findings were derived from a single-center study conducted in Shenzhen. Future prospective studies involving multi-center cohorts and diverse ethnic populations are warranted to externally validate the discriminative utility of TyG, CVAI, and their combination for identifying high-risk individuals.

IR and low-grade chronic inflammation exhibit a bidirectional pathogenic relationship (28). Hyperglycemia accelerates advanced glycation end product (AGE) formation, which directly impairs vascular endothelial function through pro-inflammatory and pro-coagulant effects (29). Concurrently, hyperinsulinemia enhances platelet aggregation, induces vasoconstriction, promotes thrombosis, and dysregulates lipid metabolism—synergistically driving sympathetic activation, renal sodium retention, hypertension, dyslipidemia, and atherosclerosis (30). Visceral adipose expansion further amplifies this cascade via inflammatory cytokine (e.g., IL-6, TNF-α) and free fatty acid release, establishing a self-perpetuating “IR–visceral obesity” cycle (31, 32).

Elevated TyG (integrating FPG and TG) and CVAI (incorporating BMI, WC, TG, and HDL-C) contribute to diabetes risk through interconnected mechanisms: TyG/CVAI elevation reflects FPG dyshomeostasis, triggering inflammatory cytokine release (IL-6, TNF-α) and oxidative stress (33). This environment accelerates pancreatic β-cell apoptosis and skeletal muscle insulin receptor desensitization, exacerbating IR progression (34). Both indices correlate with atherogenic dyslipidemia (elevated TG, LDL-C) (20, 35). Excess TG inhibits insulin signaling via impaired insulin receptor substrate 1 (IRS-1) phosphorylation and reduced glucose transporter 4 (GLUT4) translocation (20), culminating in peripheral insulin resistance.

TyG/CVAI-driven AGEs and reactive oxygen species (ROS) suppress endothelial nitric oxide synthase (eNOS) activity, diminishing nitric oxide bioavailability. The resultant endothelial dysfunction compromises pancreatic microcirculation, inducing β-cell ischemia and glucolipotoxicity—ultimately disrupting systemic glucose homeostasis (34).

## Conclusion

Elevated TyG and CVAI levels, as well as their combined use, were significantly associated with increased odds of diabetes. These findings suggest that TyG, CVAI, and their combination may serve as simple and effective tools for diabetes risk stratification, enabling targeted prevention and management strategies for high-risk populations.

## Data Availability

The original contributions presented in the study are included in the article/**Supplementary Material**. Further inquiries can be directed to the corresponding authors.
