# Supplementary material for: Joint association of triglyceride–glucose index and Chinese visceral adiposity index with prevalent diabetes: a cross-sectional study
Source: Front Endocrinol (Lausanne). 2026 Jun 26;17:1799208. doi: 10.3389/fendo.2026.1799208 (PMC13349915; doi:10.3389/fendo.2026.1799208)
Supplement: Supplementary file 1 [file DataSheet1.docx]

# Joint association of triglyceride glucose index (TyG) and Chinese visceral adiposity index (CVAI) with prevalent diabetes: a cross-sectional study

### Supplementary Material: Data Structure Description

**S1. Overview**
The dataset tota.csv ontains individual-level records for the study. Each row represents a unique participant. The file is stored in comma-separated values (CSV) format with UTF-8 encoding. The first row contains variable names, and all subsequent rows are observations. Missing values are represented as empty cells.

**S2. Variable Definitions**
All continuous variables are rounded as recorded. Categorical variables are encoded as integers; details are provided in the Coding Scheme (Section S3).

| Variable Name | Type | Unit | Description |
| --- | --- | --- | --- |
| label | String | – | Unique participant identifier |
| gender | Integer | – | 1 = Male, 2 = Female |
| age | Float | years | Age at enrolment |
| Nation | Integer | – | Ethnicity (see coding table) |
| education | Integer | – | Education level (see coding table) |
| marital_status | Integer | – | Marital status (see coding table) |
| Annual_Total_Income | Integer | – | Annual household income category (see coding table) |
| smoke | Integer | – | Smoking status (0 = never, 1 = current, etc.; verify with questionnaire) |
| Drink | Integer | – | Alcohol consumption (0 = no, 1 = yes) |
| rece | Integer | – | Receipt of any health-related intervention (0 = no, 1 = yes) |
| sleep | Float | hours | Average sleep duration per night |
| Measures_To_Control_BG_2 | – | – | (Empty in the provided sample; variable intended for blood glucose control measures) |
| Measures_To_Control_BG_3 | – | – | (Empty in the provided sample) |
| BG_Medication_Guidance | – | – | Medication guidance for blood glucose (empty in sample) |
| Control_Blood_Fat_Measures__2 | – | – | Lipid control measures (empty in sample; variable name appears twice in original data, possibly due to redundancy) |
| WH | Float | cm | Height |
| WE | Float | kg | Weight |
| WC | Float | cm | Waist circumference |
| SBP | Float | mmHg | Systolic blood pressure (average of multiple readings) |
| DBP | Float | mmHg | Diastolic blood pressure (average of multiple readings) |
| HR | Float | bpm | Heart rate (average of multiple readings) |
| FBS | Float | mmol/L | Fasting blood glucose |
| TC | Float | mmol/L | Total cholesterol |
| TG | Float | mmol/L | Triglycerides |
| HDL_C | Float | mmol/L | High-density lipoprotein cholesterol |
| LDL_C | Float | mmol/L | Low-density lipoprotein cholesterol |
| Diagnosed_Hypertension | Integer | – | Diagnosed hypertension (1 = yes, 2 = no) |
| Diagnosed_Diabetes | Integer | – | Diagnosed diabetes (1 = yes, 2 = no) |
| Measures_To_Control_BP_2 | – | – | Blood pressure control measures (empty in sample) |
| Measures_To_Control_BP_3 | – | – | Blood pressure control measures (empty in sample) |
| Diagnosis_Dyslipidemia | Integer | – | Diagnosed dyslipidemia (1 = yes, 2 = no, with many missing) |
| Control_Blood_Fat_Measures__2 | – | – | Lipid control measures (duplicate variable, empty in sample) |
| insomnia | Integer | – | Insomnia status (1 = yes, 2 = no; missing allowed) |
| WE_Change | Integer | – | Recent weight change (1 = loss, 2 = stable, 3 = gain, etc.; verify) |
| Hypertension_Family | Integer | – | Family history of hypertension (1 = yes, 2 = no, 9 = unknown) |
| Diabetes_Family | Integer | – | Family history of diabetes (1 = yes, 2 = no, 9 = unknown) |
| CHD_Family | Integer | – | Family history of coronary heart disease (1 = yes, 2 = no, 9 = unknown) |
| Apoplexy_Family | Integer | – | Family history of stroke (1 = yes, 2 = no, 9 = unknown) |
| Tumor_Family | Integer | – | Family history of malignant tumor (1 = yes, 2 = no, 9 = unknown) |
| HbA1c | Float | % | Glycated hemoglobin |
| INS | Float | μU/mL | Fasting insulin |
| grip | Float | kg | Hand grip strength |

Note: Some variables (Measures_To_Control_BG_2, Measures_To_Control_BG_3, BG_Medication_Guidance, Control_Blood_Fat_Measures__2, Measures_To_Control_BP_2, Measures_To_Control_BP_3) were largely empty in the provided sample. They were retained in the dataset for completeness; their definitions should be confirmed with the original data collection instrument.

**S3. Coding Scheme for Categorical Variables**
The exact coding may vary; the following table summarizes the values observed in the sample and their likely interpretations. Please refer to the original questionnaire for definitive labels.

| Variable | Observed codes | Interpretation |
| --- | --- | --- |
| gender | 1, 2 | 1 = Male, 2 = Female |
| Nation | 1 | Possibly Han Chinese; verify with study protocol |
| education | 3, 5, 6, 7 | 3 = Junior high school, 5 = High school, 6 = College, 7 = University or above (tentative) |
| marital_status | 2, 5 | 2 = Married, 5 = Divorced/Widowed (verify) |
| Annual_Total_Income | 3, 4, 5 | 3 = Medium, 4 = High, 5 = Very high (ordinal categories, verify cutoffs) |
| smoke | 0 | 0 = Never smoker (other codes not present in sample) |
| Drink | 0, 1 | 0 = Non-drinker, 1 = Current drinker |
| rece | 0, 1 | 0 = No, 1 = Yes (receipt of health intervention or management) |
| Diagnosed_Hypertension | 2 | 1 = Yes, 2 = No |
| Diagnosed_Diabetes | 2 | 1 = Yes, 2 = No |
| Diagnosis_Dyslipidemia | 2 (sparse) | 1 = Yes, 2 = No |
| insomnia | 1 | 1 = Yes, 2 = No (inferred; missing allowed) |
| WE_Change | 1, 2, 3 | 1 = Weight loss, 2 = Stable, 3 = Weight gain (verify) |
| Hypertension_Family Diabetes_Family CHD_Family Apoplexy_Family Tumor_Family | 1, 2, 9 | 1 = Yes, 2 = No, 9 = Unknown/Unclear |

**S4. Example Record (First Row)**

text

label: 230301001

gender: 2 (Female)

age: 69.91 years

Nation: 1

education: 3

marital_status: 2 (Married)

smoke: 0

Drink: 1

rece: 1

sleep: 7.5 hours

WH: 155.2 cm

WE: 58.8 kg

WC: 78.25 cm

SBP: 118 mmHg

DBP: 63 mmHg

HR: 71 bpm

FBS: 5.54 mmol/L

TC: 5.11 mmol/L

TG: 0.63 mmol/L

HDL-C: 1.61 mmol/L

LDL-C: 3.14 mmol/L

Diagnosed_Hypertension: 2 (No)

Diagnosed_Diabetes: 2 (No)

insomnia: 1

WE_Change: 2

family histories (9/9/9/9/9)

HbA1c: 5.6%

INS: 9.784 μU/mL

grip: 19.1 kg

**S5. Usage Notes**

The file can be read using statistical software (e.g., read.csv in R, pandas.read_csv in Python).

Empty cells denote missing data; no special missing value codes (such as -999) are used.

For analyses using categorical variables, refer to Section S3 for coding and consider converting to factors.

The variable Control_Blood_Fat_Measures__2 appears twice in the header row. Researchers should verify and rename if necessary before analysis to avoid conflicts.

# Supplementary Table S1 Association of TyG and CVAI with prevalent diabetes after excluding participants using antidiabetic, antihypertensive, or lipid-lowering medications

| **Exposure** | **total** | **Cases (%)** | **OR (95% CI)** | ***P* value** |
| --- | --- | --- | --- | --- |
| TyG |  |  |  |  |
| < 8.61 | 5213 | 32 (5.04%) | Ref |  |
| ≥ 8.61 | 4626 | 263 (7.93%) | 5.65 (3.87–8.51) | <0.001 |
| CVAI |  |  |  |  |
| < 45.03 | 2835 | 88 (0.60%) | Ref |  |
| 45.03≤ CVAI < 79.89 | 2654 | 124 (1.30%) | 1.00 (0.55–1.87) | 0.994 |
| 79.89≤ CVAI < 111.27 | 2303 | 135 (3.40%) | 1.59 (0.88–3.03) | 0.138 |
| ≥ 111.27 | 2047 | 173 (8.00%) | 2.38 (1.19–4.91) | 0.016 |
| Combined TyG and CVAI |  |  |  |  |
| TyG< 8.61&CVAI < 45.03 | 2536 | 8 (0.30%) | Ref |  |
| TyG< 8.61&45.03 ≤ CVAI < 79.89 | 1761 | 11 (0.70%) | 0.92 (0.36–2.44) | 0.859 |
| TyG< 8.61&79.89 ≤ CVAI < 111.27 | 948 | 7 (0.90%) | 0.72 (0.24–2.16) | 0.560 |
| TyG< 8.61&111.27 ≤ CVAI | 480 | 6 (1.70%) | 0.73 (0.21–2.45) | 0.614 |
| TyG ≥ 8.61&CVAI < 45.03 | 332 | 10 (3.00%) | 8.32 (3.22–22.17) | <0.001 |
| TyG ≥ 8.61&45.03 ≤ CVAI < 79.89 | 1079 | 24 (2.30%) | 3.65 (1.65–8.92) | 0.002 |
| TyG ≥ 8.61&79.89 ≤ CVAI < 111.27 | 1816 | 71 (4.60%) | 4.36 (2.03–10.54) | <0.001 |
| TyG ≥ 8.61&111.27 ≤ CVAI | 2071 | 158 (9.40%) | 5.31 (2.28–13.68) | <0.001 |

Abbreviations: OR, odds ratio; CI, confidence interval; TyG, triglyceride-glucose index; CVAI, Chinese visceral adiposity index; BMI, body mass index; SBP, systolic blood pressure; DBP, diastolic blood pressure.

Model adjusted for age, gender, education level, marital status, smoking status, drinking status, BMI, hypertension, dyslipidemia, frequent exercise, SBP, and DBP (equivalent to Model 3 in the main analysis).

Participants using antidiabetic, antihypertensive or lipid-lowering (n =1656) medications were excluded from this analysis.

# Supplementary Table S2 Association of TyG and CVAI with prevalent diabetes after excluding extreme values of BMI, TG, and FPG (beyond 3 SD from mean)

| **Exposure** | **total** | **Cases (%)** | **OR (95% CI)** | ***P* value** |
| --- | --- | --- | --- | --- |
| TyG |  |  |  |  |
| < 8.61 | 5692 | 122 (2.14%) | Ref |  |
| ≥ 8.61 | 5331 | 559 (11.24%) | 3.77 (3.04–4.72) | <0.001 |
| CVAI |  |  |  |  |
| < 45.03 | 2868 | 26 (0.60%) | Ref |  |
| 45.03≤ CVAI < 79.89 | 2840 | 89 (1.30%) | 1.97 (1.25–3.22) | 0.005 |
| 79.89≤ CVAI < 111.27 | 2764 | 218 (3.40%) | 3.48 (2.16–5.78) | <0.001 |
| ≥ 111.27 | 2551 | 348 (8.00%) | 4.67 (2.69–8.33) | <0.001 |
| Combined TyG and CVAI |  |  |  |  |
| TyG< 8.61&CVAI < 45.03 | 2536 | 14 (0.60%) | Ref |  |
| TyG< 8.61&45.03 ≤ CVAI < 79.89 | 1761 | 34 (1.90%) | 1.70 (0.90–3.35) | 0.112 |
| TyG< 8.61&79.89 ≤ CVAI < 111.27 | 948 | 46 (4.90%) | 2.56 (1.34–5.15) | 0.006 |
| TyG< 8.61&111.27 ≤ CVAI | 480 | 30 (6.20%) | 2.15 (1.00–4.76) | 0.053 |
| TyG ≥ 8.61&CVAI < 45.03 | 332 | 12 (3.60%) | 6.75 (3.01–14.87) | <0.001 |
| TyG ≥ 8.61&45.03 ≤ CVAI < 79.89 | 1079 | 55 (5.10%) | 6.11 (3.39–11.71) | <0.001 |
| TyG ≥ 8.61&79.89 ≤ CVAI < 111.27 | 1816 | 172 (9.50%) | 7.21 (4.04–13.81) | <0.001 |
| TyG ≥ 8.61&111.27 ≤ CVAI | 2071 | 318 (15.40%) | 8.11 (4.26–16.38) | <0.001 |

Abbreviations: OR, odds ratio; CI, confidence interval; TyG, triglyceride-glucose index; BMI, body mass index; TG, triglyceride; FPG, fasting plasma glucose; SD, standard deviation; SBP, systolic blood pressure; DBP, diastolic blood pressure.

Model adjusted for age, gender, education level, marital status, smoking status, drinking status, BMI, hypertension, dyslipidemia, hypertensive drug use, lipid-lowering drug use, SBP, and DBP (equivalent to Model 3 in the main analysis).

Participants with BMI, TG, or FPG values exceeding 3 standard deviations from the mean were excluded (n excluded = 472 ).

# Table S3 Sensitivity analysis of the joint association of TyG and CVAI with prevalent diabetes after re-modeling TyG and CVAI as continuous variables

| **Exposure** | **OR (95% CI)** | ***P* value** |
| --- | --- | --- |
| TyG (per 1 unit) | 4.00 (3.49–4.59) | <0.001 |
| CVAI (per 1 unit) | 1.02 (1.01–1.02) | <0.001 |
| TyG (per 1 SD) | 2.47 (2.26–2.71) | <0.001 |
| CVAI (per 1 SD) | 2.17 (1.78–2.64) | <0.001 |
| TyG (per 1 unit)† | 4.36 (3.74–5.11) | <0.001 |
| CVAI (per 1 unit)† | 0.99 (0.99–1.00) | 0.021 |
